# Orientation to the sun by animals and its interaction with crypsis

**DOI:** 10.1111/1365-2435.12481

**Published:** 2015-06-20

**Authors:** Olivier Penacchio, Innes C. Cuthill, P. George Lovell, Graeme D. Ruxton, Julie M. Harris

**Affiliations:** ^1^ School of Psychology and NeuroscienceUniversity of St AndrewsSouth StreetSt AndrewsFifeKY16 9JPUK; ^2^ School of Biological SciencesLife Sciences Building24 Tyndall AvenueBristolBS8 1TQUK; ^3^ Division of PsychologySocial and Health SciencesAbertay UniversityDundeeDD1 1HGUK; ^4^ School of BiologyDyers BraeUniversity of St AndrewsSt AndrewsFifeKY16 9THUK

**Keywords:** body orientation, camouflage, countershading, crypsis, thermal melanism, thermoregulation, ultraviolet protection

## Abstract

1. Orientation with respect to the sun has been observed in a wide range of species and has generally been interpreted in terms of thermoregulation and/or ultraviolet (UV) protection. For countershaded animals, orientation with respect to the sun may also result from the pressure to exploit the gradient of coloration optimally to enhance crypsis.
2. Here, we use computational modelling to predict the optimal countershading pattern for an oriented body. We assess how camouflage performance declines as orientation varies using a computational model that incorporates realistic lighting environments.
3. Once an optimal countershading pattern for crypsis has been chosen, we determine separately how UV protection/irradiation and solar thermal inflow fluctuate with orientation.
4. We show that body orientations that could optimally use countershading to enhance crypsis are very similar to those that allow optimal solar heat inflow and UV protection.
5. Our findings suggest that crypsis has been overlooked as a selective pressure on orientation and that new experiments should be designed to tease apart the respective roles of these different selective pressures. We propose potential experiments that could achieve this.

## Introduction

Orientation with respect to the sun has been observed in a wide range of species. This is often interpreted in terms of thermoregulation and/or ultraviolet (UV) protection; but in countershaded species, it may also result from pressure to exploit the gradient of coloration optimally to enhance crypsis. In this study, we consider how the angle between sun and animal affects each of these different possible drivers and demonstrate that crypsis is likely to be a more important component of why animals orient with respect to the sun than previously appreciated.

It is well known that variation in direct sunlight can influence microhabitat choice, and this can be a key for thermoregulation especially in ectotherms (Heinrich 1993; Angilletta 2009). Sunlight is also fundamental to the visual sense: many predators use vision to find their prey; thus, prey should be selected to be less visible. One means of doing this is to avoid brightly lit environments where predators’ vision will function best (Endler 1987; Endler & Thery 1996). Camouflage is another fundamental adaptation to subvert detection by visual predators and has been the subject of intense research interest (reviewed by Stevens & Merilaita 2009, 2011). We will use computational modelling to predict how orientation with respect to the sun influences crypsis offered by countershaded coloration. First, we will briefly review the camouflage literature exploring how orientation and crypsis might be linked and then other literature that posits alternative explanations for why animals orient with respect to the sun.

Many animals are darker on the part of the body that is typically exposed to a greater light intensity and lighter on the opposite side, a pattern of coloration called countershading (Thayer 1896, 1909). Countershading is widespread in the animal kingdom (see Rowland 2009 for a review). One proposed function is camouflage (Poulton 1890; Thayer 1896, 1909; Kiltie 1988; Ruxton, Sherratt & Speed 2004; Rowland 2009; Kamilar & Bradley 2011; Allen *et al*. 2012). The hypothesized camouflage benefits of countershading are threefold. First, the species may be consistently viewed against different backgrounds from above and below, for example for an aerial animal, a dark substrate vs. the light sky (Wallace 1889; referred to as background matching, BM). Secondly, countershading may conceal shadows created on the body by directional light that might otherwise be deleterious to matching the background (self‐shadow concealment, SSC, Cott 1940). A third function is obliterative shading (OS), where countershading conceals three‐dimensional form, otherwise revealed by the shading on a uniformly coloured body (Thayer 1896). Humans strongly rely on shading as a shape cue (Gibson 1979; Todd & Mingolla 1983; Langer & Bülthoff 2001; Lovell, Bloj & Harris 2012). Birds have been shown to derive shape from shading (Cook *et al*. 2012). Any visual system that relies on shape from shading can potentially be fooled by countershading.

**Figure 1:**
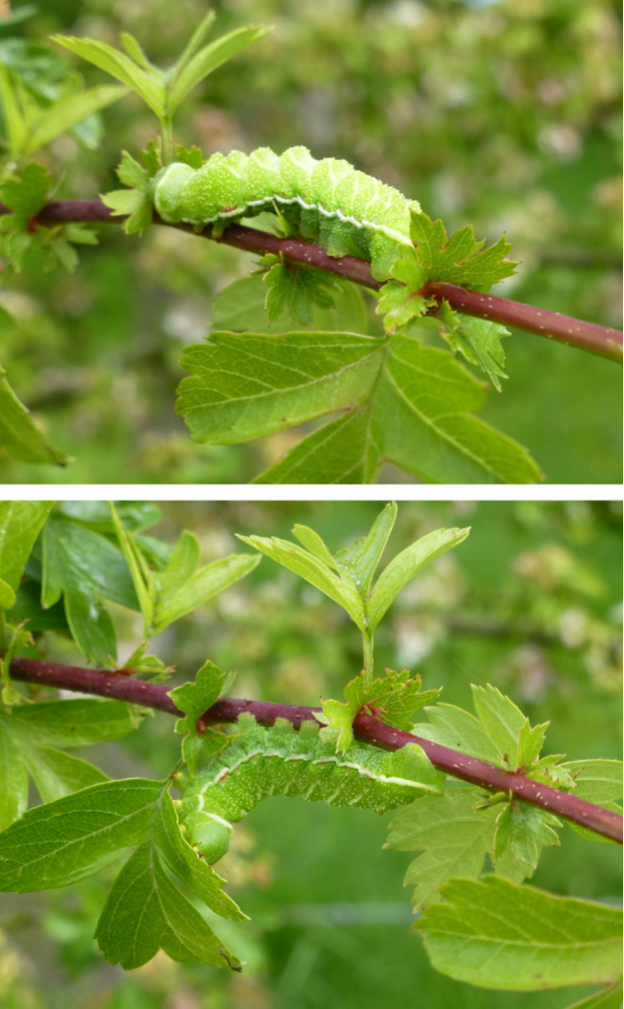
*Aglia tau* caterpillar (Tau emperor) in inverted position (back uppermost, top) and in its usual position (upside down, bottom) under the same lighting conditions (copyright of the author, Creative Commons Attribution‐Share Alike 3.0).

The mechanisms underlying these three potential functions exploit the complex interplay between light distribution and body geometry, and we have described them previously (Penacchio *et al*. 2015). One general property is that their efficiency depends on body orientation. Natural light environments are directional: most of the light comes from above and is unevenly distributed, irradiance directly from the sun being orders of magnitude greater than from other directions (Endler 1993; Darula & Kittler 2008). Consequently, a pattern of coloration that achieves BM, SSC or OS for a given body position will not be uniform, resulting in the gradation in lightness observed in so many species. Such a coloration may deliver BM, SSC or OS for a given body orientation, but could fail for different orientations. Thayer (1909, plate XII) illustrated this in paintings of an *Actuis luna* caterpillar, hanging upside down from plants, its natural position, and with its back uppermost (see Fig. 1 for a living version). In the normal position, the gradient of coloration counterbalances that of incoming light: the caterpillar is difficult to detect among the foliage (bottom). In the inverted position, gradients summate, and the caterpillar is very conspicuous (top).

Although Thayer's example is instructive, no single pattern provides a general solution to BM, SSC or OS for a wide variety of body orientations. This problem is complicated further because light distribution varies with time of the day, time of the year and weather. Accordingly, the best orientation for the body to take to reduce visibility will vary through the day and year (Penacchio *et al*. 2015). Our first objective in this study was to use computational models to predict how animals can best achieve crypsis via countershading by combining control of their orientation with respect to the sun, with their fixed surface coloration.

Other explanations for why animals orient to the sun have also been put forward. Orientation with respect to the sun has often been interpreted as a way to achieve thermoregulation (e.g. Whitman 1987; see Table S1, Appendix S3, for a review of the recent literature). Specifically, when the sun is not directly overhead, orientating with the long axis of the body parallel to the direction of the sun's rays minimizes exposure to the sun and thus the radiative energy absorbed. In contrast, a perpendicular orientation will maximize surface area and radiative heat load.

Pigmentation will also affect thermoregulation by influencing the absorption of radiation (Braude *et al*. 2001). The sun's rays most commonly strike dorsal parts of an animal, so darkening upper parts of the body could maximize heat gain from the sun and result in countershading. Note that a darker skin or pelt is not always associated with greater solar heat load (Lustick, Adam & Hinko 1980; Walsberg 1983; Dawson, Webster & Maloney 2013). Dark coloration on the back may also result from selection through protection from UV radiation (Braude *et al*. 2001). Both thermoregulation and UV protection may be better achieved through a uniform (dark) pigmentation. However, countershading allows for possible behavioural control of thermoregulation. The second aim of this study was to compare camouflage‐driven selection pressures with these alternative sun‐related selective pressures.

We begin by briefly reviewing theoretical considerations on the computation of optimal coloration for camouflage (Penacchio *et al*. 2015). We then explore how the effectiveness of a given cryptic pattern is modified when the orientation departs from optimal. Using the same computational setting, we assess how UV irradiation and solar thermal inflow depend on orientation. Our modelling focuses on terrestrial environments.

We start by choosing an optimal coloration for crypsis, for a given orientation, and then estimate the UV transmitted through the skin for all possible orientations in space. Next, we analyse the interplay between chosen orientation and solar heat. As no single model can account for the complex relationship between solar heat inflow and body coloration (Lustick, Adam & Hinko 1980; Walsberg 1983; Dawson, Webster & Maloney 2013), we propose two extreme views: (i) where pelt darkness has no influence on solar heat inflow balance and (ii) where pelt darkness drives solar heat inflow balance (i.e. thermal melanism). Finally, we discuss potential complement or conflict of the three selective pressures considered.

## Models and results

We first consider how pigmentation should be distributed across the body to maximize crypsis. We then investigate how performance declines as orientation deviates from that maximum, and when the light distribution is modified. We next examine separately how UV protection and thermoregulation fluctuate with body orientation. Finally, we explore the consequences of the interaction of these mechanisms for body colouring.

### Optimal Countershading for Crypsis: Dependence on Orientation

The optimal coloration to enhance crypsis through BM, SSC and OS varies with many factors including body shape and position, the distribution of light and the background reflectance. Although notionally different mechanisms, SSC and OS converge in their effect: they are both fulfilled when the reflectance pattern on the body provides a constant radiance, a property which is also required for BM (Penacchio *et al*. 2015). Here, when we refer to optimal reflectance for crypsis we mean a reflectance pattern that provides a flat (constant) radiance. As per Penacchio *et al*. (2015), the complex interaction between the body, the light distribution and the environment was controlled in a simulated 3D world, which allows for realistic lighting environments, using the software ‘Radiance’^1^ (Ward 1994; Radiance 2013; validated by Ruppertsberg & Bloj 2006). Within this world, we compute the irradiance impinging upon the body at different locations on the earth, different times of the day and year and for different lighting conditions (weather) (see CIE 2003; Darula & Kittler 2008). For objects that reflect light diffusely (Lambertian: objects that have a matte, rather than glossy, appearance), the relation between the irradiance falling on an infinitesimal patch on the body at location *x*,* irr*(*x*), its reflectance, *refl*(*x*), the proportion of incident radiant light reflected by the body and the radiance outgoing from the body, *rad*(*x*), which determines its appearance, is expressed by (Johnsen 2002; Fleishman, Leal & Sheenan 2006):

(eqn 1) $$rad\left(x\right)=\frac{1}{\pi }irr\left(x\right)refl\left(x\right).$$

Accordingly, once *irr*(*x*) is known, it is straightforward to determine the optimal countershading for BM, OS and SSC, by choosing the reflectance such that its product with the irradiance is constant. Then, the body appears flat and does not provide any three‐dimensional information via shape from shading. All the optimal patterns used in this study are determined using eqn (eqn 1).

**Figure 2:**
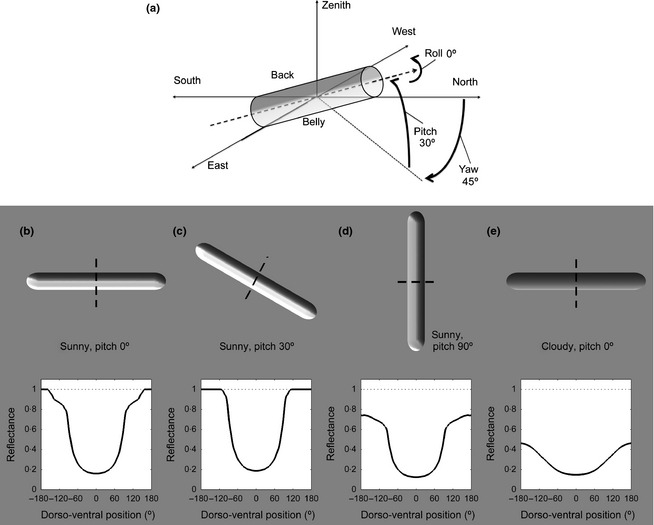
(a) Body orientation is described using yaw (−180° to 180°) and pitch (0° to 180°). Pitch values above 90° correspond to a roll of 180° (i.e. body upside down). The cylinder sits back uppermost with yaw = 45°, pitch = 30° and roll = 0°. (b–e) Influence of body position and light distribution on the optimal pattern. The light distribution corresponds to June 21, noon, St Andrews, Scotland (56° 20′25·44″N, 2° 47′43·8″W), with a standard CIE sunny sky (sun elevation 60°) for b, c and d, and a standard CIE cloudy sky in e. Top row: optimal coloration for a cylindrical body with yaw = 0° (i.e. long axis towards north), roll 0° (back uppermost) and pitch is (b, e) 0°, (c) 30° and (d) 90°. For each, the body is observed by a viewer looking west–east. Bottom row: corresponding coloration along a dorsoventral transect of the body; 0° = top of the dorsum. The reflectance of the background is 0·175.

To illustrate the principle, we considered a cylindrical body, but the method can be generalized to any body shape (see Penacchio *et al*. 2015). The orientation in space of a cylindrical body can be described by its yaw, pitch and roll (see Fig. 2a). To reduce the dimensionality of the problem, we only consider two values of roll, 0° (back uppermost) and 180° (upside down). For simplicity, we describe orientations with roll = 180° as having pitch values above 90°. Figure 2 shows how the optimal coloration of a cylindrical body varies with body orientation for one light distribution (panels b, c, d), and varies with light distribution for a single orientation (panels b, e). We show profiles of reflectance along a circular transect described by an angle $x\in \left[-180^{\circ },180^{\circ }\right]$, where *x* = 0^∘^ corresponds to the top of the dorsum. In Figs 2b–d, optimal profiles (yaw = 0°, pitch = 0°) exhibit a strong gradient of reflectance from the back to belly to compensate for the gradient of irradiance from the sunny sky. The gradient is stronger in Fig. 2c (pitch = 30°), as the back of the cylinder is oriented perpendicular to the sun (elevation = 60° at chosen latitude) and hence receives maximal irradiance. The gradient of coloration is more moderate for a cloudy sky (Fig. 2e) than for a sunny sky (Fig. 2b).

Using this model, it is evident that a particular patterning may be optimal for crypsis for a given body orientation and a specific light distribution but may fail for others, as illustrated in Figs 1 and 2.

We computed to what extent patterning and orientation combinations are suboptimal (that is, how quickly camouflage is lost). For a specific light distribution and body orientation, we determined the optimal coloration for camouflage and then computed the departure from delivering a flat radiance for other orientations.

It is unclear how departure from optimality should be measured. Flatness can be characterized physically without reference to the viewer's visual system. In contrast, finding a measure that quantifies the strength of shading *perception* requires modelling of the predator visual system. Instead, Tankus & Yeshurun (2009) proposed an operator for the detection of three‐dimensional convex objects from a computer vision perspective and used it as a measure of detectability of shading patterns. For a cylindrical body and a natural distribution of light (peaks in only one direction), the standard deviation of the outgoing radiance of a body transect satisfactorily captures departure from flatness. Thus, if $refl^{\theta_{0}}\left(x\right)$ is the optimal reflectance for the cylindrical body for reference orientation θ_0_, the departure from optimality when the body assumes orientation θ is

(eqn 2) $$d\left(\theta \right)=\mathrm{std}\left(irr^{\theta }\left(x\right)refl^{\theta_{0}}\left(x\right)\right),$$

where $irr^{\theta }\left(x\right)$ is the irradiance falling on the body for orientation θ. When θ = θ_0_, the patterning counterbalances shadowing and *d* = 0.

**Figure 3:**
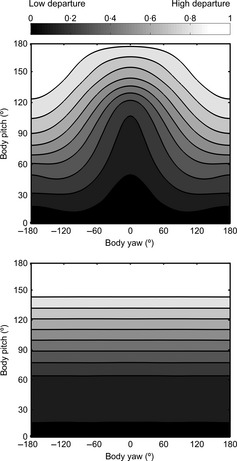
Departure from a flat radiance profile, *d*, for suboptimal orientation decisions. Reference orientation θ_0_ (yaw = 0°, pitch = 0°, roll = 0°), St Andrews, Scotland, June 21, noon (summer solstice, sun azimuth = 180°, sun elevation = 60°), with (top) sunny weather and (bottom) cloudy weather. Heat maps show deviation from flat radiance (black), as per eqn (eqn 2) and normalized into [0, 1], for pitch vs. yaw. Light colour represents high deviation, and dark colour represents low deviation. The plots are normalized separately.

Figure 3 shows how a given coloration, optimal to deliver a flat radiance for a chosen light distribution and orientation, departs from optimality when the orientation of the body deviates from the reference. We computed the irradiance impinging upon the body for a number of different orientations and then determined the radiance outgoing from the body using eqn (eqn 1). We next computed to what extent the radiance deviated from being constant (eqn (eqn 2)). Dark values in Fig. 3 correspond to a flat radiance profile (low values of *d*) whereas light values correspond to high departure from optimality. The reference orientation, yaw = 0° and pitch = 0°, is the same for the two panels of Fig. 3, notice that the heat map is darkest (*d* = 0) for these values. For a sunny sky (top panel), the irradiance distribution is highly directional. Departure from optimal is mild for orientations when the darkest part of the body (here, the back) is roughly directed towards the directions of strongest irradiance, namely the direction of the sun and the geographical zenith. For a cloudy sky (bottom panel), the light distribution only varies with pitch as the downwards irradiance does not depend on the sun direction. Thus, so far, we can conclude that there will be more departure from optimality when animals are viewed under sunny skies.

**Figure 4:**
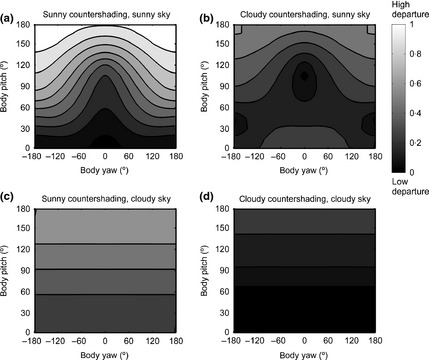
Deviation from optimal camouflage with change in orientation and/or lighting condition. Time of the day, time of the year, geographical location match those of Fig. 3, top panel. Each row is data from a specific sky (sunny, cloudy), and each column is a particular optimal countershading (for sunny or cloudy conditions). The four departure plots have been normalized jointly to have a global maximum departure of 1.

A pattern of colour may be optimal only for a specific light distribution. Figure 4 displays heat maps showing how suboptimality varies with both changes in orientation and light distribution. The top row (a, b) shows deviations for a sunny sky and body orientation (yaw = 0°, pitch = 0°), but with countershading optimal for sunny (a) or cloudy (b) weather. The bottom row (c, d) shows deviations for the same conditions but a cloudy sky. Notice that the graphs in (b) and (d) have very few dark regions. This means that no orientation decision leads to perfect camouflage when there is a mismatch between the actual light distribution and the light distribution a pattern is optimal for. Nevertheless, some orientations (e.g. low body pitch, yaw close to zero) are more likely to deliver close to optimal camouflage.

For a cloudy sky (d), the radiance outgoing from the body has less variation and hence provides fewer cues to the shape of the body, as illustrated by the overall darker values (best camouflage) in (d) in comparison with (a). This difference illustrates that departure from perfect camouflage is less important when it is cloudy.

To conclude this section, we have shown that, to maximize crypsis, optimal countershaded patterns can be found for given traits of the individual and environmental circumstances. Deviation of the organism from the optimal orientation with respect to the sun causes a significant drop‐off in these benefits; this drop‐off is less dramatic under cloudy conditions or other low light conditions (e.g. a thick canopy).

### UV Protection: Dependence on Orientation

Dark coloration patterns, generally caused by the presence of melanin, can serve as UV protection. Melanin acts as protection for the organism by preventing oxidation damage through the formation of free radicals induced by the penetration of UV radiation (Mason, Ingram & Allen 1960; Brenner & Hearing 2008). In humans, exposure to UV radiation in natural environments is a strong predictor of skin reflectance (Jablonski & Chaplin 2000).

In this section, we describe work in which we used our computational model to determine the irradiance falling upon a body. This allowed us to compute the irradiation of the body in the UV range. Using this information, we explore how patterns of skin reflectance that achieve optimal countershading for crypsis can best combine with body orientation to offer the highest UV projection and again how quickly performance deviates from that optimum with perturbations in orientation.

The UV radiation that penetrates the atmosphere is mainly composed of UVA (320–400 nm) and UVB (280–320 nm). UVC radiation (200–280 nm) is blocked by the atmosphere before reaching the earth's surface. We focused our analysis on UVB as its contribution to DNA photodamage is orders of magnitude greater than that of UVA (Brenner & Hearing 2008). Shorter wavelength light scatters more than longer wavelength light in the atmosphere, and, in spite of the enormous contribution of direct sunlight to the total downwards irradiance, the contribution of skylight is considerable at the shorter wavelengths. In the UVA range, skylight contributes 25–50% of the total irradiance, and this rises to 50–100% in the UVB range (Johnsen 2012). The distribution of UVB irradiance therefore has two main components: a strongly directional one that peaks in the direction of the sun and a more uniform one coming from the hemispherical sky. Thus, the distribution of damaging UV radiation is not as biased towards the direction of the sun as generally assumed (see Johnsen 2012). To account for the spatial and spectral differences of these two components, we started by separating the irradiance into two parts: irradiance coming directly from the sun, *irr*_sun_, and from the sky, *irr*_skylight_. It is possible to achieve this spatial separation because the CIE functions underlying the model of skylight in the ‘Radiance’ program make this distinction (CIE 2003; Darula & Kittler 2008). To compute the relative contribution from sunlight and skylight to irradiance at different wavelengths, we used standard descriptions of solar and skylight irradiance (American Society for Testing Materials 2008) and converted values from watts to photons (see Johnsen 2012). The distribution of irradiance then reads

(eqn 3) $$irr\left(x,\lambda \right)=irr_{\mathrm{sun}}\left(x,\lambda \right)+irr_{\mathrm{skylight}}\left(x,\lambda \right),$$

where λ denotes wavelength. We model the transmittance of the integument, which governs the fraction of light that passes through it, as a product of the transmittances of distinct anatomical layers, one of which is composed of melanin. We assume that changes in body reflectance only affect the composition of the melanin layer. Assuming that the quantity of melanin is inversely proportional to the reflectance of the pelt, *refl*(*x*), and proportional to the thickness Δ of the anatomical layer it defines, the absorbance *a* of the skin or pelt reads

(eqn 4) $$a\left(x,\lambda \right)\propto A\left(\lambda \right)\left(1-refl^{\theta_{0}}\left(x\right)\right)\Delta ,$$

where *A*(λ) is the spectral absorbance of melanin, in m^−1^, and $refl^{\theta_{0}}\left(x\right)$ is the optimal patterning for orientation θ_0_. Thus, the total quantity of UVB absorbed by an infinitesimal patch at location *x* on the body, with orientation $\theta ,R_{\mathrm{UVB}}^{\theta }\left(x\right)$, is expressed by

(eqn 5) $$R_{\mathrm{UVB}}^{\theta }\left(x\right)\propto \int_{\lambda \;\mathrm{in}\;\mathrm{UVB}}\;e^{-a(x,\lambda )}irr^{\theta }\left(x,\lambda \right)d\lambda$$

Note that eqn (eqn 5) provides the quantity of radiation only up to a constant factor. This is not a problem for our purposes, since this factor is constant across the body. The spectral absorbance of melanin we use in the calculations comes from Kollias (1995).

**Figure 5:**
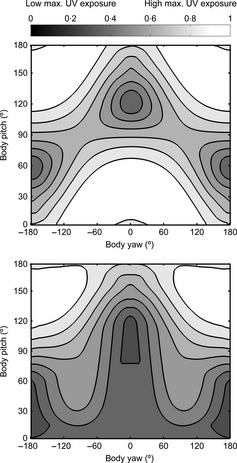
Dependence on orientation of relative UVB exposure for a cylindrical body with (top) a white integument and (bottom) a countershaded coloration. Time of the day, time of the year, geographical location match those of Fig. 3, top panel. The thickness of the melanin layer Δ was set to (top) 0 m, that is no melanin at all, and (bottom) 0·001 m for the computations. Both plots are normalized jointly to have an overall maximum irradiation of 1.

We assessed how the relative exposure to UVB radiation varies with body orientation (Fig. 5). A reference orientation θ_0_ was first chosen which yielded an optimal coloration for camouflage $refl^{\theta_{0}}\left(x\right)$, as explained in section ‘Optimal Countershading for Crypsis: Dependence on Orientation’. We then determined both the spectral irradiance due to direct sunlight and to skylight falling on the body for a large set of orientations spanning the set of all possible body orientations. We computed the relative quantity of radiation transmitted through the skin according to eqn (eqn 5). For each body orientation θ, the computation provided the relative quantity of radiation that penetrates an infinitesimal transect of the body through an infinitesimal patch at location *x* along a transect of the body as $R_{\mathrm{UVB}}^{\theta }\left(x\right)$. As UVB radiation penetrates and acts locally in the body, it makes sense to minimize the maximum quantity of radiation exposure across the whole body. Accordingly, we determined the maximal quantity of radiation transmitted to the inner part of the body through an infinitesimal patch by taking the maximal values of $R_{\mathrm{UVB}}^{\theta }\left(x\right)$ over *x* across the transect determined by orientation θ, namely:

(eqn 6) $$I_{\mathrm{UVB}}^{\max}\left(\theta \right)=\max_{x\;\mathrm{in}\left[-180^{\circ },180^{\circ }\right]}R_{\mathrm{UVB}}^{\theta }\left(x\right).$$

Figure 5 displays heat maps showing how $I_{\mathrm{UVB}}^{\max}$ varies with orientation for (top) a skin or pelt with no melanin (the animal has a uniform light coloration) and (bottom) when countershaded (dark on top, light below) with a melanin layer responsible for its coloration. In the top panel, maximal exposure to UVB is minimal (black in figure) for orientations where the long axis of the body is parallel to the direction of the sun's rays (sun elevation = 60°). This orientation has yaw = 0°, pitch = 120° or, equivalently, yaw = ±180°, pitch ≈ 60°, depending whether the body is back uppermost or upside down.

In contrast, irradiance for the reference orientation (yaw = 0°, pitch = 0°) and contiguous orientations, where optimal camouflage is achieved, is very high as the back of the cylinder is nearly perpendicular to the direction of the sun. This is illustrated by there being a region near pitch = 0°, yaw = 0° which is white in Fig. 5 (top), hence far from optimal. Thus, crucially, the orientation offering best UV protection (low maximal exposure, dark in Fig. 5) offers very poor camouflaging via countershading and vice versa.

When the body is countershaded, that is when melanin is present on the dorsal side, UVB irradiance is strongly reduced for orientations in which the dorsal side is oriented upwards. This is shown in Fig. 5 (bottom plot). Compare the wide light grey‐white region around yaw = 0°, pitch = 0° in the top plot (no melanin), with the bottom plot, which contains no such region. In particular, melanin lessens UVB irradiation for the reference orientation (yaw = 0°, pitch = 0°). It is worth noting, however, that even if the part of the body with maximal melanin directly faces the sun, UVB exposure is still at a minimum (darkest values) when the body's long axis is directed towards the sun: this can be observed in Fig. 5, bottom plot, by comparing the black areas around yaw = 0°, pitch = 120° and yaw = ±180°, pitch = 60° (long axis in direction of the sun) and the lighter values at yaw = 0°, pitch = 0° (reference orientation, the dorsal surface faces the direction of the sun). This occurs because the relative contribution of skylight to downwards UVB light is strong, even for low elevations, and hence, UVB radiation enters from a lateral direction, corroborating the idea that the distribution of damaging UV is not as biased towards the sun as commonly thought (Johnsen 2012). Of course, a fully melanic coloration (where the animal is completely dark, not countershaded) would offer a better protection against UVB than the countershading pattern for any orientation.

In conclusion, the novel consideration of body orientation with respect to light distribution offered by this computational model allows us to show that the influence of orientation on UV irradiation has two main features. (i) The best behavioural way to minimize UV irradiation is to have the long axis of the body aligned with the direction of the sun. (ii) As most UV incident on an animal will be from above, assuming dark pigmentation is costly, we would expect that protection for UV would select for a countershaded patterning. Thus, at first sight, it seems likely that both crypsis and UV protection share benefits from countershaded patterning. However, there is conflict between the two mechanisms in terms of the way behaviour combines with patterning, as the orientations offering best UV protection (yaw = 0°, pitch = 120°, yaw = ±180°, pitch 60°) offer very poor camouflaging via countershading and, vice versa, the orientations offering best camouflaging (yaw and pitch close to 0°) are not optimal for UV protection (compare Figs 4 and 5). We will explore the potential consequences and resolution of this conflict in section ‘Combining Mechanisms’ below.

### Thermoregulation: Link Between Body Coloration and Orientation

Body orientation may influence thermal exchange in different ways. Here, we focus on the interplay between body reflectance and thermoregulation through radiative heat flow, exploring how thermoregulatory selection pressures might impinge on exterior patterning and orientation behaviour.

Thermoregulation through solar thermal exchange is more complex than commonly understood, and depends on pelt/plumage insulation properties, as well as colour. It is commonly thought that dark surfaces are more likely to have a greater heat gain than light surfaces when exposed to the sun, a principle referred to as thermal melanism (Clusella‐Trullas, van Wyk & Spotila 2007). According to this view, it is beneficial for an animal to orientate the darkest part of its body towards the sun only when heating is required. However, the principle that dark integuments cause greater heating than light integuments under solar exposure proves to be an oversimplification. Lustick, Adam & Hinko (1980) showed that in birds orientation may modify the qualitative relation between plumage colour and solar heat gain. Walsberg (1983) outlined the wide range of strategies possible for natural selection to accommodate the relationship between coat colour and solar thermal exchange in birds and mammals. Clusella‐Trullas, van Wyk & Spotila (2007) reviewed thermal melanism in ectotherms and reported strong evidence that melanism provides an enhanced fitness in cold climates as melanistic ectotherms generally have higher values of total energy absorbed than their lighter counterparts. In Clusella‐Trullas, van Wyk & Spotila (2009), the authors showed that the solar heating rate of melanistic lizards was higher than that of similar non‐melanistic species.

Dawson, Webster & Maloney (2013) addressed the putative conflict between thermal needs and crypsis in mammals. They showed that although the polar bear *Ursus maritimus* and koala *Phascolarctus cinereus* have very different fur colorations, their heat influx through solar radiation is similar and concluded that the lower the insulation power of the fur, the higher the influence of colour on solar heating. It is worth noting that, in some species, colour in the visible range may not correlate with a body's spectral absorption in the near‐infrared range (e.g. lizard *Uma scoparia*, Norris 1967).

In the light of these considerations, it appears that no simple model can encompass the interplay between coloration and solar heat flow. We decided to consider two different simplifications of reality, based on two extreme views. In the first (hypothesis 1), reflectance does not affect solar heat load at all. The second view (hypothesis 2), in contrast, is in line with the principle of thermal melanism that darker body colorations lead to increased solar heat load. Crucially, the novelty of both of our models is that they take into account the relative position of the body and the light distribution, a driving component of solar thermal exchange. Indeed, whatever the connection between body reflectance and solar heat load and whatever the insulation power of the pelt, orientating the body's long axis perpendicular to the sun's rays will maximize the irradiance and thus radiative heat inflow. Conversely, minimal heat load can be achieved by orienting the body's long axis parallel to the sun's rays. Thus, the orientation that offers minimal heat inflow also offers best protection from UV (see section ‘UV Protection: Dependence on Orientation’) but offers poor camouflage through countershading (see section ‘Optimal Countershading for Crypsis: Dependence on Orientation’). Conversely, orientation that maximizes heat load also has the potential to offer maximal crypsis but also maximizes exposure to the potential for UV damage.^2^

Under the first hypothesis, given a specific body pelt, there is no relation between absorbance at visible wavelengths and solar heat load. Accordingly, only the irradiance impinging on the body should be taken into account. Thus, for body orientation θ, the solar heat load through an infinitesimal transect is

(eqn 7) $$Q_{\mathrm{Hyp}1}\left(\theta \right)\propto \int_{-180^{\circ }}^{180^{\circ }}irr^{\theta }\left(x\right)dx,$$

where the irradiance is decomposed into its direct and diffuse components, $irr\left(x,\lambda \right)=irr_{\mathrm{sun}}\left(x,\lambda \right)+irr_{\mathrm{skylight}}\left(x,\lambda \right)$,whose relative contributions in watts are computed using standard descriptions of solar and skylight irradiance (American Society for Testing Materials 2008) and integrated over the infrared range 700–2500 nm.

Under the second hypothesis, solar heat flow follows the rule of thermal melanism that higher absorbance in the visible range, that is lower reflectance, provides a higher solar heat load. Therefore, both the irradiance arriving at the body and its reflectance should be taken into account when computing solar heat exchange. In that case, for reference orientation θ_0_ and the corresponding optimal coloration $refl^{\theta_{0}}\left(x\right)$, the solar heat load is given by

(eqn 8) $$Q_{\mathrm{Hyp}2}\left(\theta \right)\propto \int_{-180^{\circ }}^{180^{\circ }}irr^{\theta }\left(x\right)\left(1-refl^{\;\theta_{0}}\left(x\right)\right)dx.$$

Our modelling is simplified, as we have not considered the effects of fur, or made the assumption that irradiance and reflectance in the visible spectrum can be generalized to the whole spectrum. However, the modelling is valuable because it takes into account the relative position of the body and the light distribution.

Our model allows the prediction of preferred choices of orientation for a given purpose (either exploiting or minimizing heat load from the sun).

**Figure 6:**
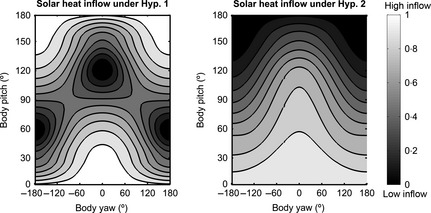
Relative solar heat flow for to hypothesis 1 (left panel) and 2 (right panel). Lighting conditions (i.e. sunny sky at noon, sun elevation 60°), orientation and coloration of the cylindrical body are the same as in Fig. 3, top panel. A Light colour represents high thermal inflow, and a dark colour represents low thermal inflow (heat exchange in the two panels are normalized between 0 and 1, independently).

How solar heat inflow varies with orientation according to the first hypothesis is illustrated in Fig. 6 (left). Lighter values correspond to higher solar heat inflow. Thermal inflow is at a minimum when the long axis of the body is directed towards the sun (yaw = 0°, pitch = 120°, or yaw = ±180°, pitch 60°). Here, the direction of the strongest component of the downward irradiance is perpendicular to the body transect and thus has least effect according to the cosine law of irradiance. Maximal heat inflow is obtained when the long axis of the body is perpendicular to sun's rays (yaw = 0°, pitch = 30°).

Compare Fig. 6 (left) with Fig. 5 (top), showing UVB irradiance for a body with no layer of melanin. In both, the main body orientation feature driving solar heat load is the angle between the long axis of the body and sun's rays (remember, the sun's azimuth is 180° and elevation is 60°; thus, the direction of maximal irradiance occurs for points on the plots with coordinates yaw = 0° and pitch = 120° or, equivalently, yaw = ±180° and pitch = 60°.)

Under the second hypothesis, of thermal melanism, heat inflow is maximum when the back of the body, its darkest part, is perpendicular to sun's ray (yaw = 0°, pitch = 30°, Fig. 6, right). This means it is mainly regulated by the position of the darkest part of the body with respect to the sun.

Taken together, introducing body orientation into the modelling of solar thermal exchange allows us to draw the following conclusions. In thermal melanism, two features drive radiative heat flow, namely the orientation of the darkest part of the body towards the sun and the overall orientation of the body with respect to the perpendicular to sun's rays. Only the second feature drives radiative heat flow if thermal melanism is not assumed, resulting in a very different prediction for what would be optimal (see Fig. 6 left and right).

### Combining Mechanisms

All three selective pressures, camouflage, UV protection and thermoregulation, predict a strong dependence on orientation behaviour. Here, we explore the potential compatibility of orientation behaviours driven by these three pressures, to understand whether it is possible to distinguish between which is at work in the natural environment.

So far, we used a single reference orientation (yaw = 0°, pitch = 0°, roll = 0°) and a single position of the sun (azimuth 180°, elevation 60°), and assumed that the long axis of the body at the reference yaw and the sun azimuth are aligned. In Appendix S1 (Supporting Information), we show that the conclusions we draw below on the interaction between the three selective pressures are not specific to these choices.

We now analyse whether optimal orientations for the three functions are similar. First note that it is not easy to link decreases in crypsis, heat load or UV protection directly to quantitative changes in fitness. Therefore, only qualitative results in terms of comparison of optimal loci within the orientation space are possible.

#### Animals that can assume ‘any’ orientation in space

Let us first assume that the body can take any orientation in space. If thermal melanism is not assumed (hypothesis 1, section ‘Thermoregulation: Link between Body Coloration and Orientation’), crypsis and thermoregulation both show a high dependence on reference pattern orientation. This happens because thermoregulation through solar radiation is primarily driven by the angle between the long axis of the body and a plane perpendicular to the sun direction. As orientations perpendicular to the sun maximize solar heat inflow, the predictions for best thermoregulation (maximum heat inflow) and crypsis are similar, as long as the optimal countershading is for body axis orientations close to perpendicular to the sun's rays (compare the regions with yaw around 0° and pitch around 30° in Fig. 1, top, and Fig. 6, left).

Orientation with respect to this perpendicular is also central for the hypothesis of thermal melanism (hypothesis 2, section ‘Thermoregulation: Link between Body Coloration and Orientation’). However, now another component contributes. Body orientations that deliver optimal crypsis (darkest part of the body faces a greater light intensity) provide a higher radiative heat flow (Fig. 3, top, and Fig. 6, right). Thus, orientations that make a countershaded pattern best cancel shadowing, increase heat inflow and may help partially compensate for the impossibility of orienting the body perpendicularly to the sun's rays on angled substrates.

Overall, whether radiative heat exchange follows the rules of thermal melanism (hypothesis 2) or not (hypothesis 1), countershading camouflage, is compatible with thermoregulation (maximizing heat gain), provided orientations do not deviate too much from the perpendicular to sun's rays. Of course, if heating is to be avoided, the opposite conclusion may be drawn: crypsis through countershading and thermoregulation would conflict.

*Minimizing UVB exposure*: To minimize exposure to UVB, the optimal orientation depends on the sun and the zenith. Orientations for which the long axis of the body is around the zenith give rise to far less irradiation than others. Compare Fig. 5 top and bottom panels: although the melanin layer helps reduce UVB transmission, orientating the long axis of the body towards the sun (yaw ±180°, pitch 60°, or yaw 0°, pitch 120°, dark regions correspond to a low exposure to UV) still offers more protection to UVB radiation than other orientations. It is possible to compute optimal coloration for orientations that maximize both camouflage and UVB protection. However, these orientations are antagonistic to obtaining optimal heat gain through solar radiation. On the other hand, if cooling is required these orientations offer an optimal solution for the three selective pressures considered.^3^

*Optimizing crypsis*: Assume that a pattern of coloration is chosen to optimize crypsis (e.g. Fig. 3, top) and provide high radiative heat inflow. This is feasible to compare the pattern of optimality in Fig. 3, top, and the white region near yaw 0° and pitch 0° in Fig. 6 (left and right). Our simulations show that these two selective pressures are compatible with UV protection as coloration reduces UVB irradiation considerably, as shown by the reduction of irradiation for orientations around the reference orientation (yaw 0°, pitch 0°) between Fig. 5 top panel (no melanin) and bottom panel (dark coloration due to melanin). Here, even if optimal orientation decisions for countershading camouflage and thermoregulation, and UV protection, do not coincide, UV protection benefits from countershading and the same behavioural orientations fit with the three selective pressures.

To sum up, for animals that can assume any orientation in space, orientations are compatible to exploit countershading for crypsis and to favour high radiative inflow. They may not coincide with optimal orientations for UV protection, but the melanic coloration filters out UV radiation where it is at its maximum.

#### Animals limited to horizontal orientations

For animals that can only adjust their yaw, we show in Appendix S2 that orientations to optimize the countershading pattern for visual camouflage and for UV protection coincide. Orientations that favour positive solar heat balance depend on the time of the day and/or on the thermal properties of the integument. They lead to poor camouflage under hypothesis 1 (colour has no influence on thermal inflow) and under hypothesis 2 (thermal melanism) for low elevation of the sun.

### Disentangling Selective Pressures

**Figure 7:**
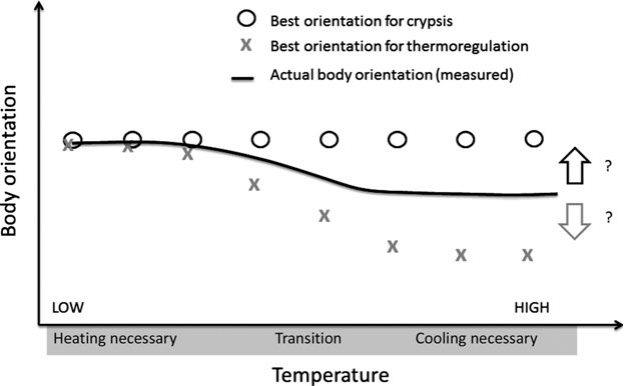
When cooling is important, and the main factor acting on heat balance is solar radiation, observation of actual body orientation may help disentangle the two pressures. If the observed orientations (black curve) are close to the optimal orientation for crypsis (circles), priority would be given to crypsis over thermoregulation. If they are close to the optimal orientation for thermoregulation (crosses), thermoregulation would be privileged.

In this section, we propose strategies to tease apart the potential role of the three selective pressures on orientation and coloration. A first proposal is to record the actual behaviour of an animal when optimal orientations for exploiting the countershading pattern for different selective purposes differ. For example, under hypothesis 2 (thermal melanism), on a sunny day when the ambient temperature is high, orientations to maximize crypsis and minimize solar heat inflow are antagonistic (see Fig. 3 top and Fig. 6 right). In such a case, the behavioural response of the animal should determine whether crypsis or thermoregulation is favoured. Figure 7 illustrates this first proposal graphically.

Our modelling used a realistic distribution of light, reminiscent of the lighting that can be found in the natural environment. However, in the laboratory, it is possible to use many light sources with different spectral properties. A second proposal is to build artificial light distributions in the laboratory where the geometrical distribution of light from light sources in distinct ranges of the spectrum (UV, infrared, visible to the animal) can be fully controlled in such a way that optimal orientations to favour two of the selective pressures contemplated are antagonistic (for example, where a source of infrared and a source of UV light are placed in opposite directions with respect to the animal). Again, the behavioural response of the animal would help determine which function is placed first. However, this proposal would only work provided the perception of the different patterns of light does not rely on the same sensory process.

## Summary and conclusions

We have considered three types of selective pressure in which animal coloration plays a central role. We have shown that orientation with respect to the sun is of primary importance for carrying out these diverse functions. We have next assessed potential conflicts between optimal orientation decisions for each of these selective pressures. For many situations, the three functions for coloration and orientation deliver predictions that largely coincide. Notable conflicts do arise, however. For example, when heat inflow is detrimental, orientating the long axis of the body in the direction of the sun both minimizes solar heat load and maximizes UV protection, but may be prejudicial to optimal camouflage (section ‘Optimal Countershading for Crypsis: Dependence on Orientation’). Further, when heat inflow is wanted, optimal UV protection and optimal heat inflow are antagonistic and only trade‐offs between optimizing coloration for these opposing purposes can be found (sections ‘UV Protection: Dependence on Orientation’ and ‘Thermoregulation: Link between Body Coloration and Orientation’). These exceptions provide a clear set of circumstances that could be tested in behavioural experiments.

Despite the exceptions outlined above, the central prediction of our modelling is that orientations to exploit the countershading pattern for crypsis, thermoregulation and UV protection are generally compatible. As a consequence, most behavioural responses to optimize orientation for these different purposes are theoretically entangled. Nevertheless, most studies on animal orientation with respect to the sun have explained orientation to the sun as a behavioural response to enhance solar heat inflow or UV protection (Waldschmidt 1980; Gonyou & Stricklin 1981; Clark & Ohmart 1985; Hofmeyr & Louw 1987; Whitman 1987; Kuntzch & Nel 1990; O'Neill, Kemp & Johnson 1990; Rocha & Bergallo 1990; Bauwens, Hertz & Castilla 1996; Gandolfi & Rocha 1998; Brown & Downs 2007). Our modelling shows that orientation to maximally exploit countershading for crypsis, by directing the darkest part of the body towards the sun, is a valid alternative selective pressure to account for observed orientation to the sun. Therefore, we argue that the role of behavioural orientation for enhancing visual camouflage may have been overlooked in the literature. Conversely, the fact that orientation behaviours evolved to gain thermoregulatory or UV protection benefits also allow for crypsis via countershading may allow the exploitation of this form of crypsis to be more widely adopted that previously assumed.

Our modelling has shown that the selective pressures on orientation with respect to the sun are not mutually exclusive, as they provide very similar predictions for optimal orientation. Is there a gradation of importance where a given selective pressure should be privileged? With this question in mind, we have proposed experiments to tease apart orientation behaviour to favour different selective pressures. However, here, we should issue a word of caution. Our models have dealt with the physics of light and have avoided any description or discussion of the sensory systems of animals. Particular sensory systems may not be sensitive to the full spectrum, may sense radiation through their indirect effect on the body (heat) and thus may be unable to disentangle the information needed to fine‐tune the preferred orientation for crypsis, thermoregulation and UV protection. Put another way, the correlation between the orientation responses to favour the three non‐exclusive selective pressures may already exist at the level of sensory processing.

Empirically, there have been diverse studies demonstrating non‐random orientation with respect to the sun for individuals of diverse animal taxa. We summarize these studies in Appendix S3, Table S1, along with the mechanisms to explain that orientation, as considered by the authors. In the overwhelming majority of studies, the authors have interpreted their results in terms of thermoregulation. However, we have emboldened entries relating to studies where, on reading the paper, we consider that crypsis and/or UV protection might also usefully be considered as potential underlying drivers of the observed orientation behaviour.

For the Arachnid studies, the orb spiders show strong orientation behaviours when sunlight is strong but air temperatures are relatively low and/or there is sufficient wind to provide convective cooling; this suggests to us that UV protection should be considered as well as the authors’ focus on thermoregulation. Further, orb spiders more generally are known to have a range of behavioural and physiological adaptations to reducing their conspicuousness both to the prey and potential predators whilst they are stationed in the centre of their webs, so we feel that greater consideration of crypsis in these particular cases is also warranted. Turning to reptiles, the Seychelles giant tortoise has very little of its body directly exposed to sunlight and very high thermal inertia, so we feel that UV protection especially of the head seems at least as plausible as the author's focal putative mechanism of thermoregulation. Lack of obvious predators means that we consider crypsis unlikely to be a strong driver of orientation behaviour in this species. However, for all of the other reptiles listed in Table S1 predation rates are known to be high, and the species have a range of behavioural responses (e.g. freezing, fleeing and vigilance) interpreted as being linked to reducing rates of contact with predators. For this reason, we think that camouflaging aspects of orientation behaviour deserve further consideration. Exactly the same arguments can be made for the highlighted mammalian studies where orientation behaviours are stronger when air temperatures are higher and wind speeds are lower, leading the authors to focus on thermoregulation as the likely driver of orientation. However, orientation with respect to the sun is still non‐random when environmental conditions suggest that thermoregulation should be less of a concern. This makes it at least plausible that UV protection and/or crypsis might also be relevant. The focal species in these studies live in open environments with strong direct sunlight and generally clear skies (increasing exposure to UV) and are known to suffer high levels of predation and to show behaviours linked to reducing exposure to predators.

In conclusion, our modelling, although a simplification of reality, grasps the main features of the interaction between orientation behaviour and crypsis, thermoregulation and UV protection. Crucially, even though the quantitative changes in fitness cannot currently be estimated, the qualitative conclusions on the interaction between the three selective pressures, based on the location of minima and maxima within the orientation space, do not depend on the accuracy of quantitative predictions. We have shown that orientations to efficiently exploit the countershading pattern to favour crypsis, thermoregulation and UV protection are mostly congruent. However, most studies on organism orientation with respect to the sun interpret orientation behaviour in terms of thermoregulation. We argue that not enough studies have contemplated crypsis as a selective pressure on orientation behaviour. We also suggest that the evolution of crypsis through countershading may be easier to understand if orientation behaviours that enhance crypsis also bring benefits through the other mechanisms discussed here.

## Data accessibility

Data are deposited in the Dryad Digital Repository: doi: 10.5061/dryad.db6 kg (Penacchio *et al*. [Link]).

## Supporting information


**LaySummary**

**Appendix S1.** Independence of the results on the choice of reference orientation.
**Appendix S2.** Compatibility of the three selective pressures for animals limited to horizontal orientations.
**Appendix S3.** Studies showing non‐random orientation with respect to the sun.

## Notes

1 Throughout, we write ‘Radiance’ with a capital letter for the software and use radiance (lower case) for the physical quantity.

2 Note that UV light is a shorter wavelength than infrared and will be scattered more by the atmosphere, so impinges on the body from a wider range of angles. The heat, from the infrared wavelengths, will have a stronger directional component (from the sun). So, for heating, the expected fall‐off will be sharper than for UV protection, but the maximum is in the same direction for both effects.

3 Of course, it is possible that a given animal might want heating at dawn, and cooling during the day, whilst maintaining countershading. However, to take such issues into account makes the problem more complex, and thus, here, we focus only on the most important features of the interaction between orientation and the three selective pressures under study.
