# Supplementary material for: Orientation to the sun by animals and its interaction with crypsis
Source: Funct Ecol. 2015 Jun 20;29(9):1165–77. doi: 10.1111/1365-2435.12481 (PMC4758631; doi:10.1111/1365-2435.12481)
Supplement: Supplementary file 1 — LaySummary [file FEC-29-1165-s001.pdf]

Functional Ecology

British Ecological Society

Orientation to the sun by animals and its interaction with crypsis

Olivier Penacchio, Innes C. Cuthill, P. George Lovell, Graeme D. Ruxton, Julie M. Harris

Many species have been shown to choose the orientation of their body with respect to the sun. A typical example of non-random orientation behaviour is the perpendicular orientation to the sun's rays adopted by cold-blooded animals such as reptiles while basking. In general, orientation to the sun has been interpreted in terms of pressure to regulate the rate of heat gain from sunlight by altering the body surface exposed to the sun's rays. Orientation to the sun has also been interpreted as a way to enhance protection from ultraviolet radiation. However, countershading, a very widespread colour pattern in the animal kingdom found in mammals, birds, fish, insects and reptiles, offers an alternative explanation for orientation to the sun. Countershaded animals show a gradient of coloration in which the part of the body that receives more light from the sun, typically the back, is darker and opposite parts are lighter. As acknowledged in 1896 by the naturalist and artist Abbott Thayer, countershading may act as visual camouflage. For countershaded animals, orientation with respect to the sun may help exploit the gradient of coloration and better balance the environmental light distribution to enhance camouflage.

We used a ray-tracing rendering software which includes ecologically realistic light environments to model how light distribution, body geometry and body coloration interact to shape the light reflected by an animal. This modelling predicted the optimal countershading pattern for camouflage. We next examined how camouflage, thermoregulation and protection from ultraviolet light fluctuate when the orientation of an animal with respect to the sun varies.

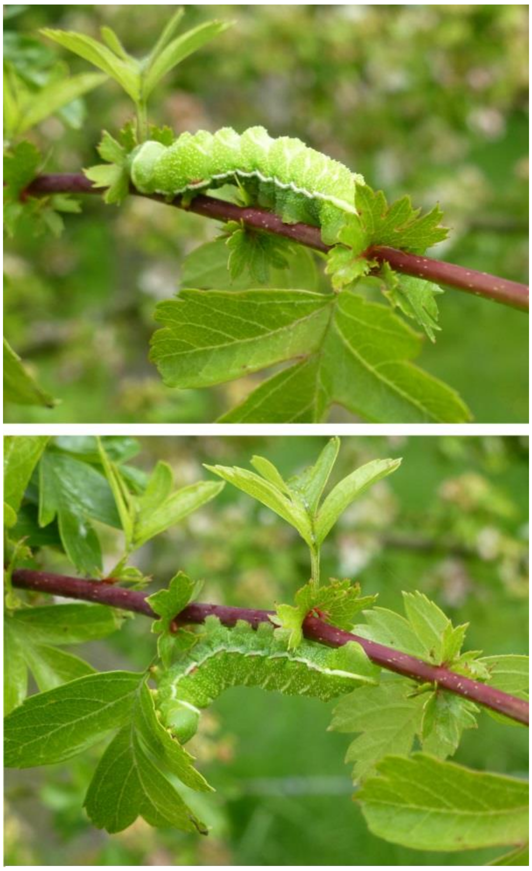

Image caption: Aglia tau *caterpillar (*Tau emperor) in *(top) inverted position (back uppermost) and (bottom) in its usual position (upside down) under the same lighting conditions. Copyright of the author (Creative Commons Attribution-Share Alike 3.0).*

Our findings show that body orientations that optimally exploit countershading for camouflage also lead to optimal solar heat gain and maximize protection from ultraviolet radiation.

To conclude, we argue that not enough studies have considered camouflage as a selective pressure on orientation behaviour. This study also suggests that the evolution of camouflage through countershading may have been promoted by the compatibility of orientation behaviour to enhance concealment with other selective pressure on orientation. We propose experiments to disentangle different selective pressures on orientation with respect to the sun.
