# Supplementary material for: Orientation to the sun by animals and its interaction with crypsis
Source: Funct Ecol. 2015 Jun 20;29(9):1165–77. doi: 10.1111/1365-2435.12481 (PMC4758631; doi:10.1111/1365-2435.12481)
Supplement: Supplementary file 2 — Appendix S1. Independence of the results on the choice of reference orientation. Appendix S2. Compatibility of the three selective pressures for animals limited to horizontal orientations. Appendix S3. Studies showing non‐random orientation with respect to the sun. [file FEC-29-1165-s002.docx]

**Supporting information**

**Mansucript title: Orientation to the sun by animals and its interaction with crypsis**

**Olivier Penacchio^a^, Innes C. Cuthill^b^, P. George Lovell^a,c^, Graeme D. Ruxton^d^, Julie M. Harris^a^**

**^a^ School of Psychology and Neuroscience, South Street, University of St Andrews, St Andrews, Fife KY16 9JP UK**

**^b^ School of Biological Sciences, Life Sciences Building, 24 Tyndall Avenue, Bristol BS8 1TQ, UK**

**^c^ Division of Psychology, Social and Health Sciences, Abertay University, Dundee, DD1 1HG, UK**

**^d^ School of Biology, Dyers Brae, University of St Andrews, St Andrews, Fife KY16 9TH UK**

**E-mail: (OP) op5@st-andrews.ac.uk**

**Appendix S1. Independence of the results on the choice of reference orientation**

Figures S1-2 below show how Fig. 3 is modified as the reference orientation is varied (compare also Figures S3, S4, S5 with Figures 4, 5, 6). No major change occurs in the relative position of the optimal orientations for cryptic countershading, UV protection and thermoregulation. This relation would only be modified if the reference orientation coincided with the direction of the sun, when all functions would be similar (assuming heating is to be avoided). Similarly, when the elevation of the sun is shifted, all the plots follow smooth deformations and the relative configuration of optimal orientations is maintained. Finally, if the sun’s azimuth and the reference yaw differ, asymmetric countershading results. We did not investigate the consequence of asymmetric patterning. Although some species can change their coloration asymmetrically (e.g., cuttlefish, Langridge 2006), no species that show evidence of orientation to the sun have asymmetric coloration. To sum up, the discussion in the main text on the interaction between the three selective pressures is not specific to our choice of a reference orientation.

**
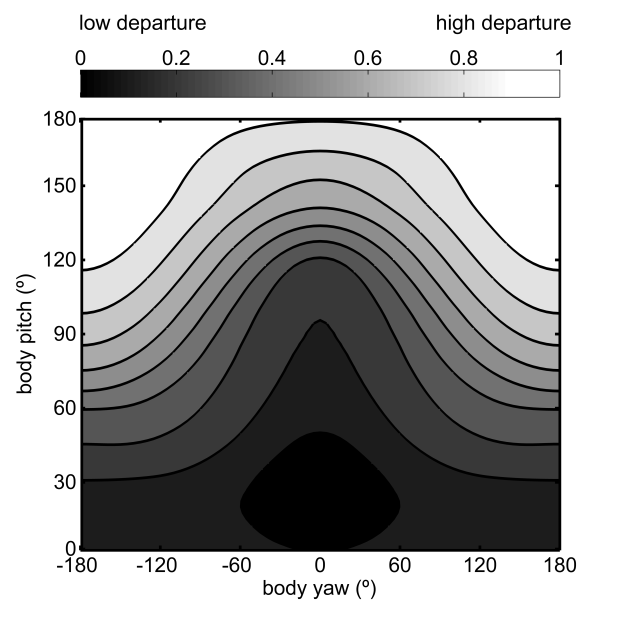
**

**
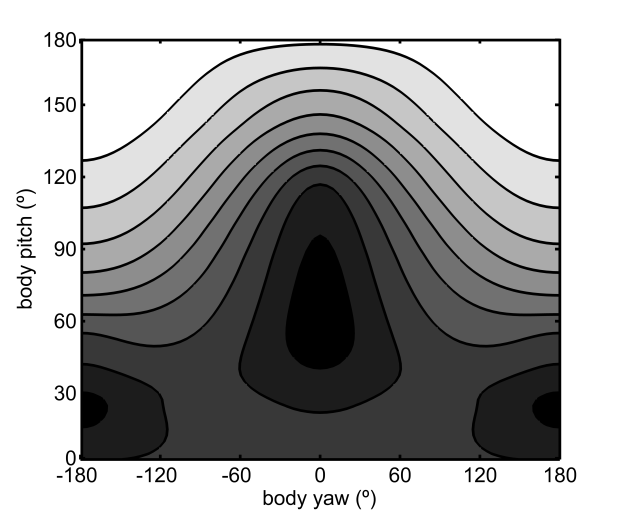
**

**
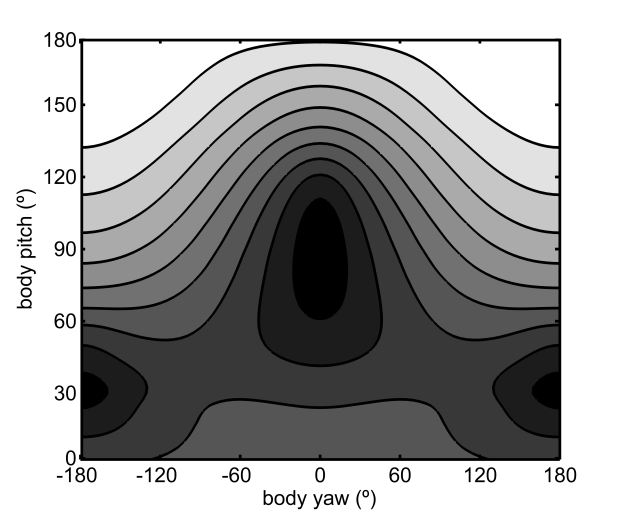
**

Figure S1. Departure from the delivering of a flat radiance with changes in orientation for sunny weather and three different values for the reference orientations, namely (top) yaw=0º and pitch=30º, (middle) yaw=0º and pitch=60º, and (bottom) yaw=0º and pitch=90º. The lighting conditions (type of sky, time of the year, time of the day) are the same as in the top panel of Fig. 3 in the main text.


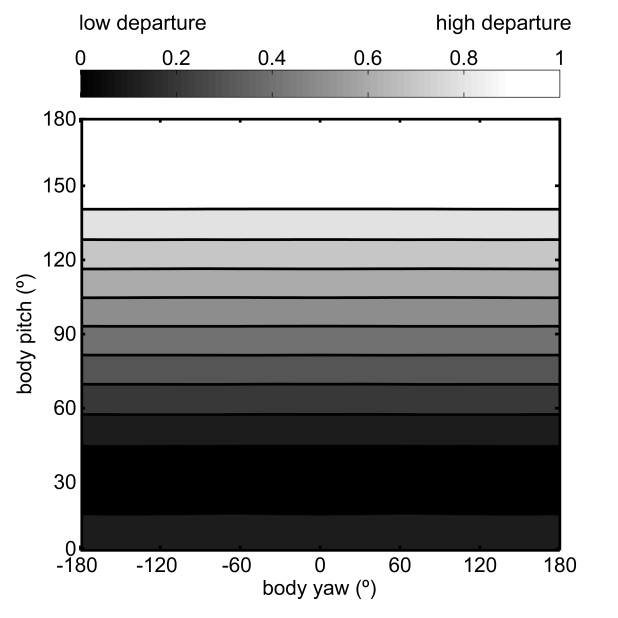


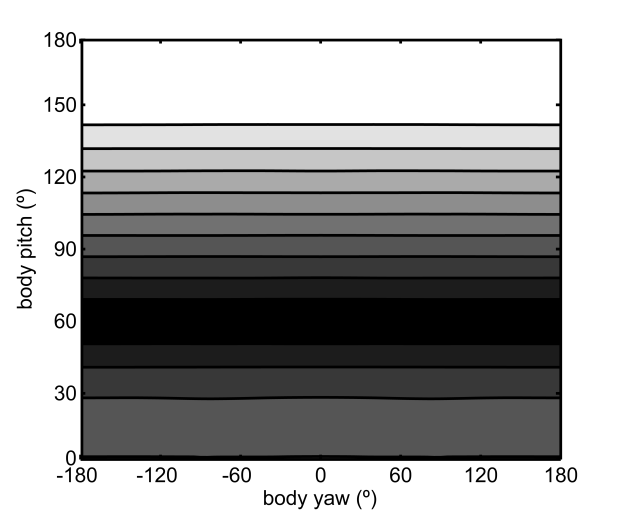


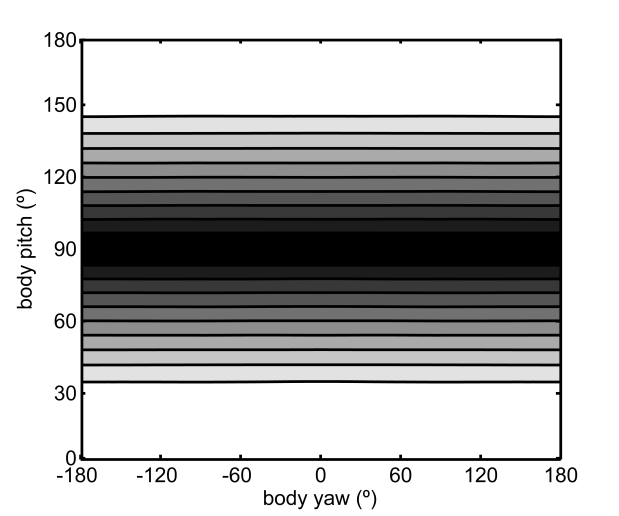


Figure S2. Departure from the delivering of a flat radiance with changes in orientation for cloudy weather and three different values for the reference orientations, namely (top) yaw=0º and pitch=30º, (middle) yaw=0º and pitch=60º, and (bottom) yaw=0º and pitch=90º. The lighting conditions (type of sky, time of the year, time of the day, geographical location) are the same as in the bottom panel of Fig. 3 in the main text.


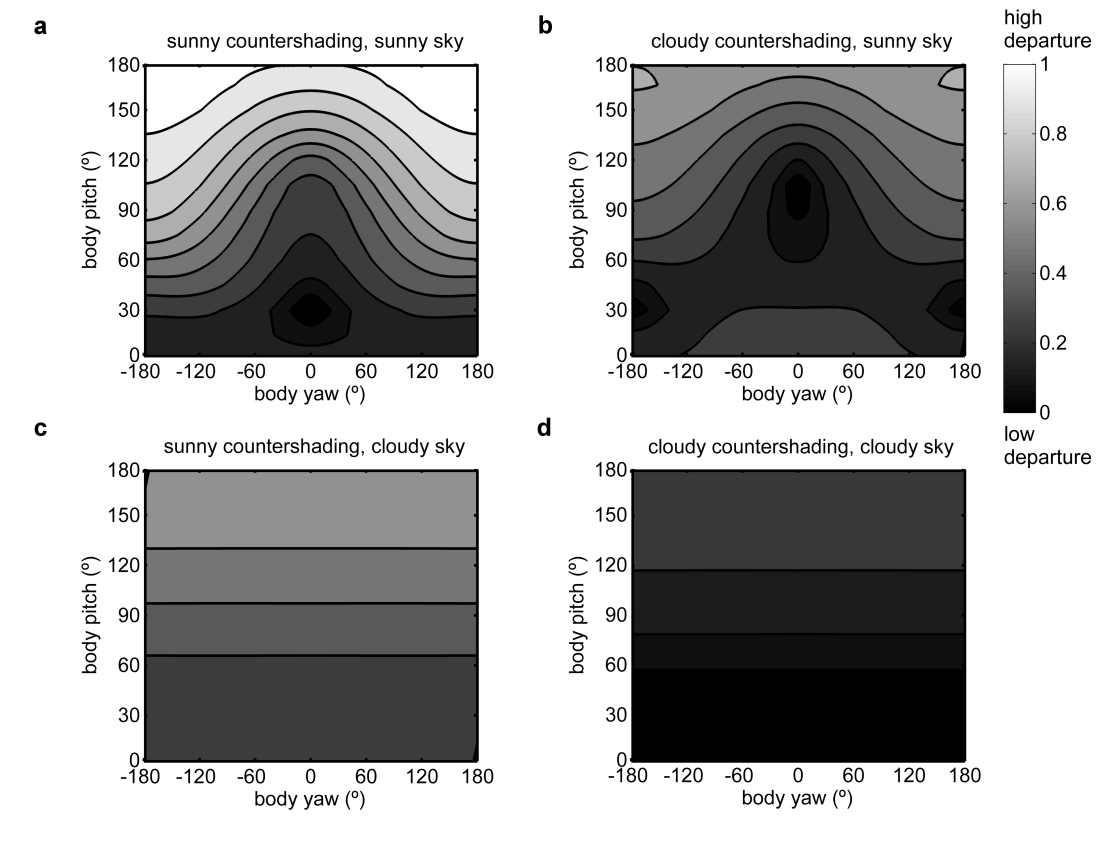


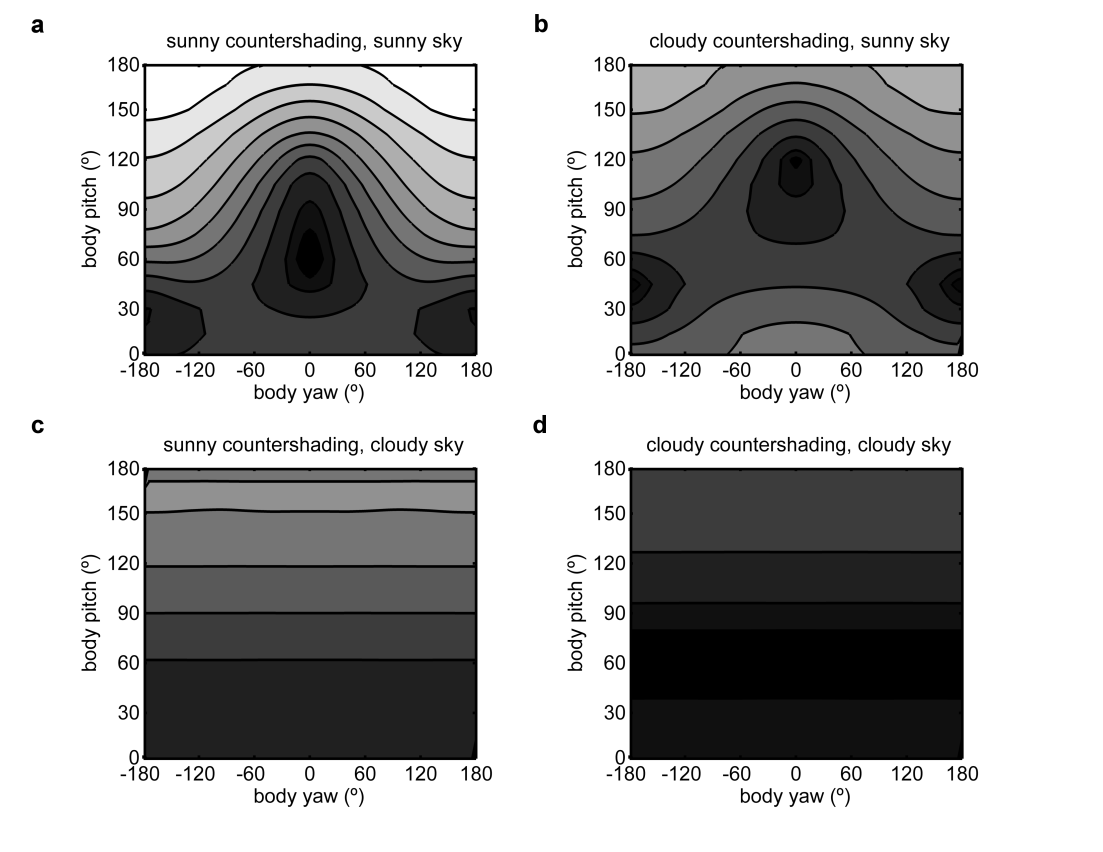


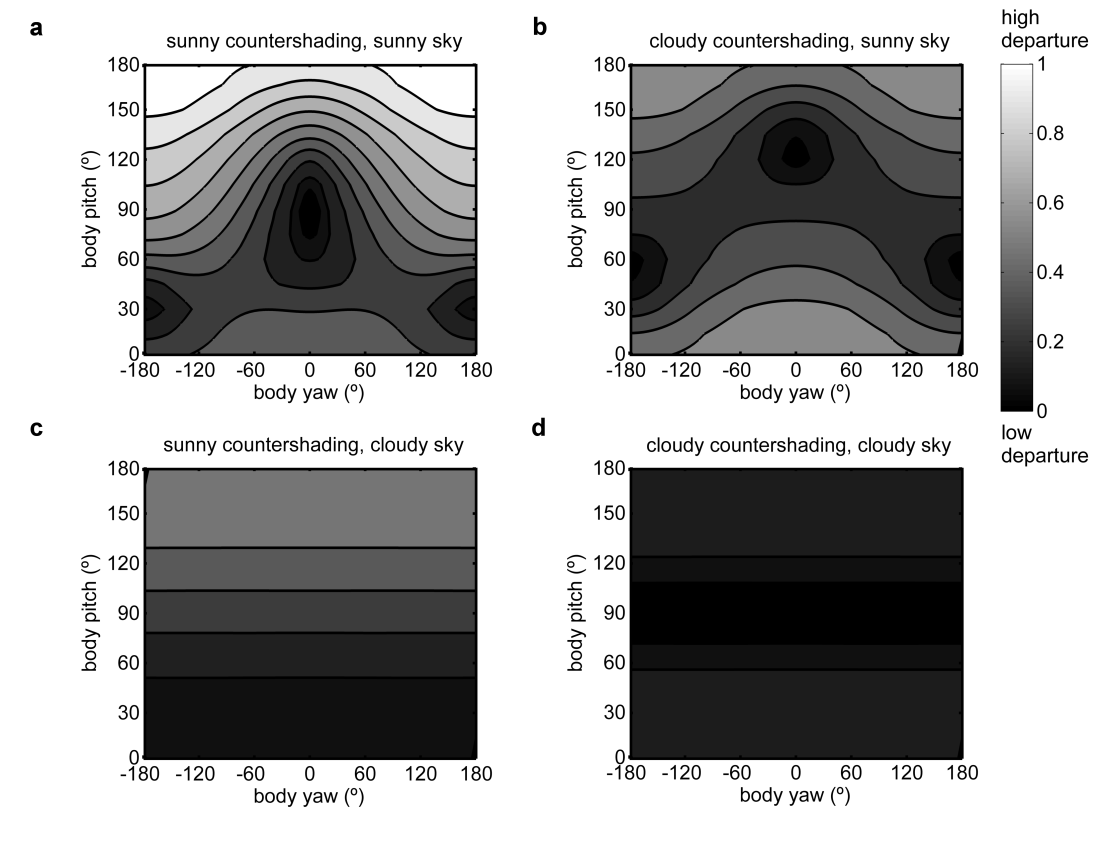


Figure S3. Deterioration of camouflage with change in orientation and/or change in lighting condition for three different values for the reference orientation, namely (top block) yaw=0º and pitch=30º, (middle block) yaw=0º and pitch=60º, and (bottom block) yaw=0º and pitch=90º. In each block, the subplots are organised as in Fig. 4 in the main text: the top row (resp., the bottom row) corresponds to a sunny sky (resp., a cloudy sky), and in the left column (resp., right column) the pattern of reflectance is optimal for a sunny sky (resp., a cloudy sky); the four departure plots of each block have been normalized jointly to have a global maximum departure of 1. All the conditions (time of the day, time of the year, geographical location) match that of Figs. 3 and 4 in the main text.


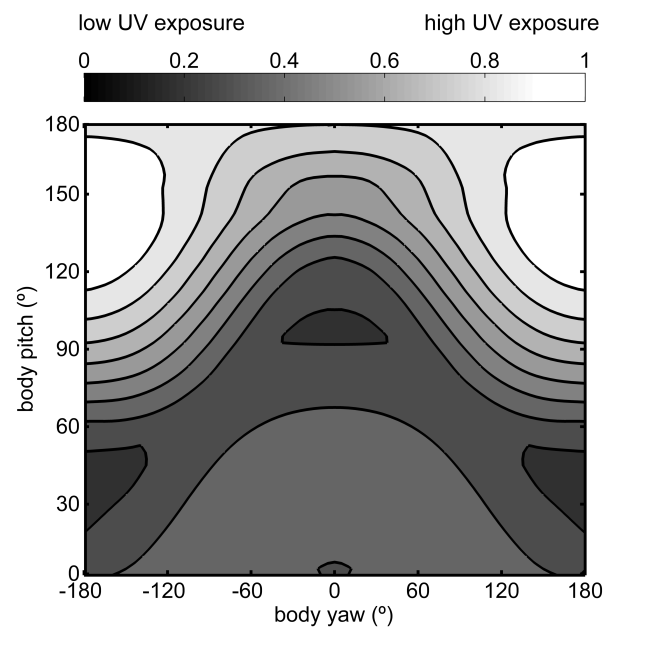


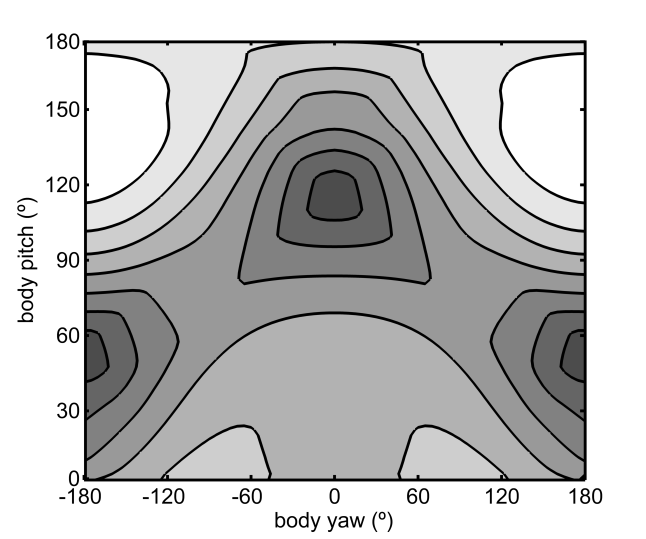


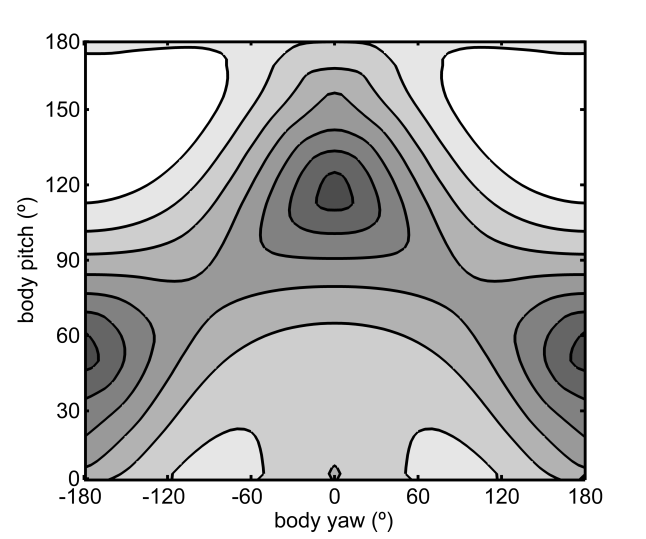


Figure S4. Dependence on orientation of relative UVB exposure for a cylindrical body with an optimal counter-shaded coloration for three different values of the reference orientation, namely (top) yaw=0º and pitch=30º, (middle) yaw=0º and pitch=60º, and (bottom) yaw=0º and pitch=90º. The lighting conditions and the parameters are the same as in Fig. 5 in the main text.


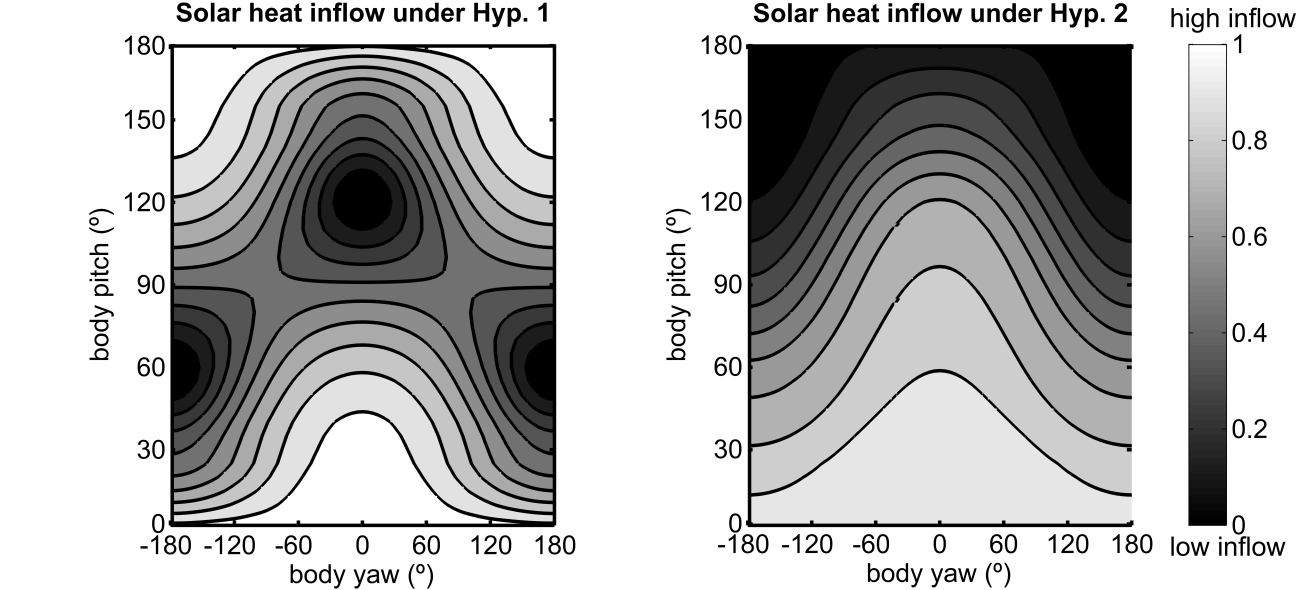


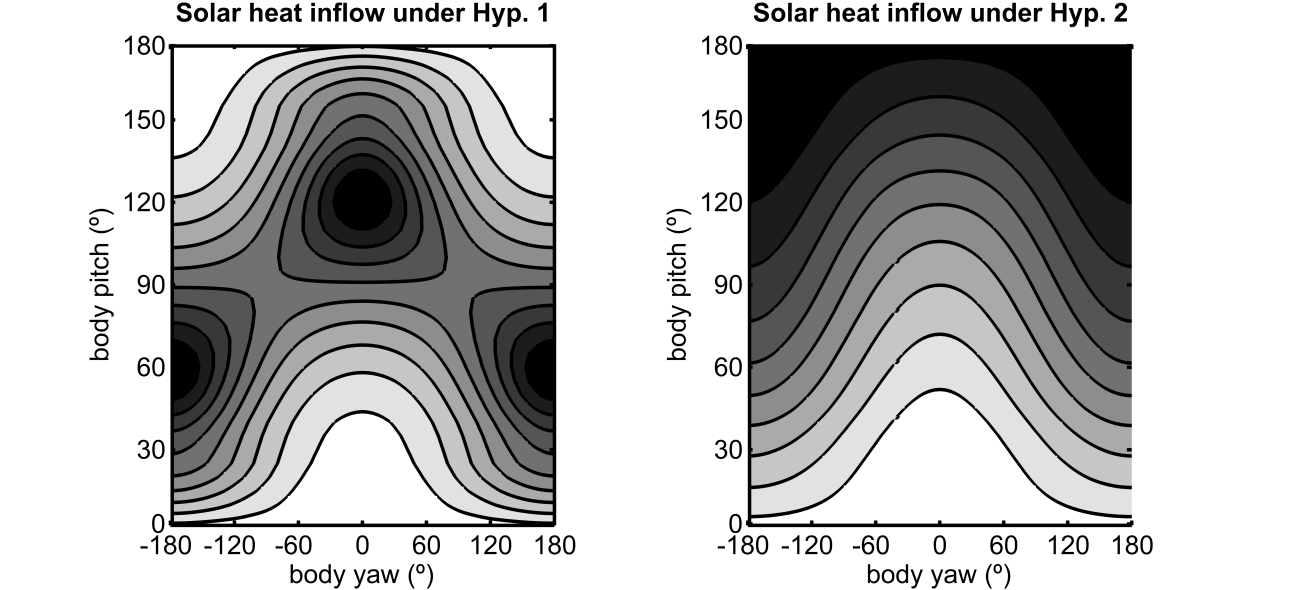


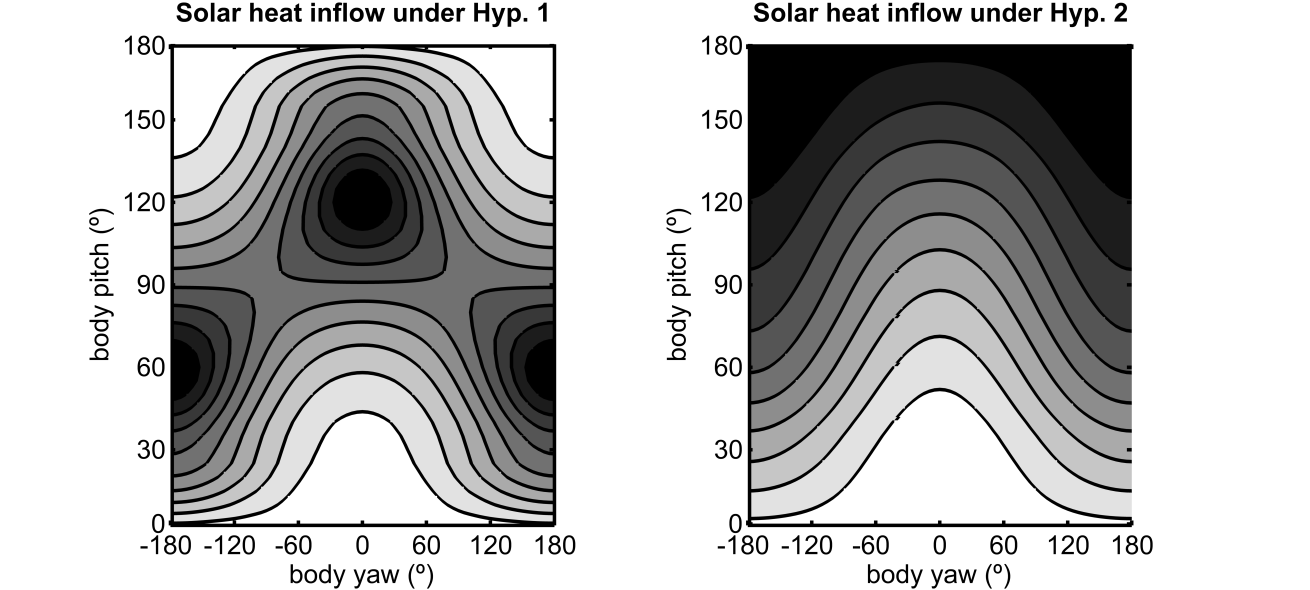


**Figure S5. Relative solar heat load according to Hypothesis 1 (left panels) and Hypothesis 2 (thermal melanism, right panels) for three different values of the reference orientation, namely (top) yaw=0º and pitch=30º, (middle) yaw=0º and pitch=60º, and (bottom) yaw=0º and pitch=90º. The lighting condition is the same as in Fig. 6 in the main text. The three left panels are identical since under Hypothesis 1 thermal exchanges do not depend on body coloration.**

**Additional reference Appendix S1**

Langridge, K. V. (2006) Symmetrical crypsis and asymmetrical signalling in the cuttlefish *Sepia officinalis*. *Proceedings of the Royal Society B-Biological Sciences*, **273**, 959-967.

**Appendix S2. Compatibility of the three selective pressures for animals limited to horizontal orientations.**

Ground- dwellers can only adjust their yaw, greatly simplifying the analysis of the compatibility between optimal orientations for the three selective pressures. Consider a body with a counter-shaded coloration. For a cloudy sky, the distribution of light is constant across azimuthal directions, so none of the selective pressures is affected by changes in yaw. For sunny weather, the countershading pattern best counterbalances the shadowing created by the distribution of light, when both the yaw of the animal and the azimuth of the sun coincide (animal faces towards or away from the sun). The same is true for the best protection against UV irradiation, thus the best orientations for crypsis and UV protection coincide for ground-dwellers.

If we suppose that body coloration has no influence on solar heat exchange (Hypothesis 1, Section C), solar heat inflow is maximum when the body long-axis and the sun’s azimuth are orthogonal and minimum when they are aligned. Consequently, in ground-dwellers, thermoregulation is *in conflict* with crypsis and UV protection under this hypothesis when *heating* is beneficial to the organism and *compatible* when *cooling* is beneficial.

Under the hypothesis of thermal melanism (Hypothesis 2, Section C), the relation between solar thermal inflow and body yaw is subordinated to the elevation of the sun. When the sun is high in the sky orientating the body long-axis in the direction of the sun maximizes solar heat inflow. Orthogonal orientations maximise heat inflow when the sun is low in the sky.

Taken together, orientations that maximise visual camouflage using the countershading pattern and minimise UVB irradiation coincide for ground-dwellers. Orientations that lead to a positive solar heat inflow depend on the elevation of the sun (time of the day) and on the thermal properties of the skin or pelt.

**Appendix S3. Studies showing non-random orientation with respect to the sun.**

**Table S1: Recent studies (ordered by class, and by date within each class) showing non-random orientation with respect to the sun, and the putative underlying mechanism or mechanisms considered by the authors. We embolden entries where we consider that crypsis and/or UV protection too might usefully be considered as underlying drivers of orientation behaviour**.

| **Class** | **Species** | **Reference** | **Suggested mechanisms** |
| --- | --- | --- | --- |
| Arachnid | *Nephila clavipes* (golden silk orb-web spider) | Robinson & Robinson 1974; Higgins & McGuinness 1991 | **thermoregulation** |
|  | *Argirope trifasciata* (orb-weaving spider) | Tolbert 1978 | **thermoregulation** |
| Gastropod | *Echinolittorina peruviana* (periwinkle) | Munoz et al. 2005 | thermoregulation |
| Insect | *Hipparchai semele* (grayling butterfly) | Findlay, Young & Finlay 1983 | thermoregulation |
|  | *Taeniopoda eques* (black desert grasshopper) | Whitman 1987 | thermoregulation |
|  | Efferia spp. (robber flies) | O’Neill, Kemp & Johnson 1990 | thermoregulation |
| Reptile | *Uta stansburiana* and *Sceloporus undulates* (iguanid lizards) | Waldschidt 1980 | **thermoregulation** |
|  | *Geochelone gigantean* (Seychelles giant tortoise) | Frazier 1988 | **thermoregulation, avoidance of glare** |
|  | *Tropidurus oreadicus* (Sauria, Iguanidae) | Rocha & Bergallo 1990 | **thermoregulation** |
|  | *Podarcis hispanica atrata* (lacertid lizard) | Bauwens et al. 1996 | **thermoregulation** |
|  | *Tropidurus torquatus* (Sauria, Tropiduridae) | Gandolfi & Rocha 1998 | **thermoregulation** |
|  | *Gallotia galloti* (Tenerife lizard) | Bohorquez-Alonzo, Font & Molina-Borja 2011 | **intraspecific signalling** |
| Birds | *Diomedea immutabilis* and *D. Nigripes* (Laysan and black-footed albratrosses) | Howell & Bartholomew 1961 | thermoregulation |
|  | *Sula dactylatra* (gannet) | Bartholomew 1966 | thermoregulation |
|  | *Spheniscus demerus* (African penguin) | Frost, Siegfied &Burger 1976 | thermoregulation |
|  | *Anhinga anhinga* (Anhinga ) | Hennemann 1982 | thermoregulation |
|  | *Anas rubripes* (American black duck) | Brodsky & Weatherhead 1983 | thermoregulation |
|  | *Cathartes aura* (Turkey vulture). | Clark & Ohmart 1985 | thermoregulation |
|  | *Parus bicolour* (tufted titmouse) and *Parus carolinesnsis* (Carolina chickadee) | Wood & Lustick 1989 | thermoregulation |
|  | *Phalacrocorax carbo* (cormorant) | Sellers 1995 | thermoregulation (plumage drying) |
| Mammals | Bos spp. (domestic cattle) | Gonyou & Stricklin 1981 | thermoregulation |
|  | *Xerus inaudis* (cape ground squirrel) | Bennett et al. 1984 | **thermoregulation** |
|  | *Antidorcas marsupilis* (springbok) | Hofmeyr & Louw 1987 | thermoregulation |
|  | *Nyctereutes procyonoides* (raccoon dog) | Harri & Korhonen 1988 | thermoregulation |
|  | Giraffa spp. (giraffe) | Kuntzch & Nel 1990 | thermoregulation |
|  | *Connachaetes gnou* (black wildebeest) | Maloney, Moss & Mitchell 2005 | thermoregulation |
|  | *Procavia capensis* (rock hyrax) | Brown & Downs 2007 | **thermoregulation** |
|  | *Connochaetes taurinus* (common wildebeest), *Tragegelaphus oryx* (eland) and *Aepyceros melampus* (impala) | Hetem et al. 2011 | **thermoregulation** |

**Additional reference Appendix S3**

Bartholomew, G.A. (1966) The role of behaviour in the temperature regulation of the masked booby. *Condor*, **68**, 523-535.

Bennett, A.F., Huey, R.B., John-Alder, H. & Nagy, K.A. (1984) The parasol tail and thermoregulatory behaviour of the cape ground squirrel *Xerus inauris*. *Physiological Zoology*, **57**, 57-62.

Bohorquez-Alonzo, M.L., Font, E. & Molina-Borja, M. (2011) Activity and body orientation of *Gallotia galloti* in different habitats and daily times. *Amphibia-Reptilia*, **32**, 93-103.

Brodsky, L.M. & Weatherhead, P.J. (1983) Behavioural thermoregulation in wintering black ducks: roosting and resting. *Canadian Journal of Zoology*, **62**, 1223-1226.

Frazier, J. (1988) Orientation of giant tortoises *Geochelone gigantean* Schweigger while grazing on Aldabra Atoll. *Amphibia-Reptilia*, **9**, 27-32.

Frost, P.G.H., Siegfied, W.R. & Burger, A.E. (1976) Behavioural adaptations of the Jackass penguin, *Spheniscus demersus* to a hot, arid environment. *Journal of Zoology*, **179**, 165-187.

Harri, M. & Korhonen, H. (1988) Thermoregulatory significance of basking behaviour in the raccoon dog (*Nyctereutes procyonoides*). *Journal of Thermal Biology*, **13**, 169-174.

Hennemann, W.W. (1982) Energetics and spread-wing behaviour of anhingas in Florida. *Condor*, **84**, 91-96.

Higgins, L. & McGuinness, K. (1991) Web orientation by *Nephilia clavipes* in Southern Texas. *American Midland Naturalist*, **125**, 286-293.

Howell, T.R. & Bartholomew, G.A. (1961) Temperature regulation in Laysan and black-footed albatrosses. *Condor*, **63**, 185-187.

Munoz, J.L.P., Finke, G.R., Camus, P.A. & Bozinovic, F. (2005) Thermoregulatory behaviour, heat gain and thermal tolerance in the periwinkle *Echinolittorina peruviana* in central Chile*. Comparative Biochemistry and Physiology A*, **142**, 92-98.

Robinson, M.H. & Robinson, B.C. (1974) Adaptive complexity - thermoregulatory postures of the golden-web spider, *Nephila clavipes*, at low latitudes. *American Midland Naturalist*, **92**, 386-396.

Sellers, R.M. (1995) Wing-spreading behaviour of the cormorant *Phalacrocorax carbo*. *Ardea*, **83**, 27-36.

Tolbert, W.W. (1979) Thermal stress of the orb-weaving spider *Argiope trifasciata* (Araneae). *Oikos*, **32**, 386-392.

Wood, J.T. & Lustick, S.L. (1989) The effects of artificial solar radiation on wind-stressed tufted titmice (*Parus bicolour*) and Carolina chickadees (*Parus carolinensis*) at low temperatures. *Comparative Biochemistry and Physiology*, **92**, 437-477.
